# Effect of docosahexaenoic acid and olive oil supplementation on pup weight in alcohol-exposed pregnant rats

**DOI:** 10.3389/fped.2024.1334285

**Published:** 2024-04-04

**Authors:** Deepak Yadav, Enrique M. Ostrea, Charlie T. Cheng, Esther Kisseih, Krishna R. Maddipati, Ronald L. Thomas

**Affiliations:** ^1^ Department of Pediatrics, Wayne State University School of Medicine, Detroit, MI, United States; ^2^ Bioactive Lipids Research Program, Wayne State University School of Medicine, Detroit, MI, United States

**Keywords:** fetal alcohol spectrum disorders, prenatal alcohol exposure, docosahexaenoic acid (DHA), olive oil, fetal weight, inflammatory cytokines, placental weight

## Abstract

**Background:**

Low birth weight has been observed in offspring of alcoholic mothers due likely to unresolved inflammation and oxidative injury. Dietary lipids play a role in inflammation and its resolution. The primary objective was to investigate the effect of DHA and olive oil on the birth weight of pups born to alcohol-exposed dams.

**Methods:**

Pregnant rats were randomized to the control or three treatment (alcohol) groups. From gestational days (GD) 8–19, the control group received daily olive oil and malto/dextrose, whereas groups 2 and 3 received olive oil and low-dose alcohol or high-dose alcohol, respectively. Group 4 received daily DHA and high-dose alcohol. The dam's blood was collected on GD 15 and 20 for cytokine analysis. Dams were sacrificed on GD 20. The mean birth weight of pups was compared by one-way ANOVA with post hoc Duncan's test.

**Results:**

There was a significant increase in the pups' mean birth weight in the high-dose alcohol/DHA and high-dose alcohol/olive oil. Higher pro-inflammatory cytokines (IL-1β and IL-12p70) were noted in the alcohol-exposed dams.

**Conclusions:**

DHA and olive oil supplementation in alcohol-exposed pregnant rats significantly increased their pups' birth weight despite having high pro-inflammatory cytokines. The mechanism of this effect remains to be determined.

## Introduction

Alcohol use during pregnancy in humans is a significant public health problem that can result in a spectrum of fetal abnormalities known as Fetal Alcohol Spectrum Disorders (FASD), including Fetal Alcohol Syndrome (FAS) (1, 2). Despite public health warnings, 10%–15% of pregnant women drink alcohol (3, 4), with about 3% of pregnant women engaging in binge drinking, according to the CDC. The estimated prevalence of FAS varies between reports, and it's thought to occur in 0.3–0.8 per 1,000 children in the United States and in 2.9 per 1,000 globally (5). The FAS remains a significant health problem since it is a leading cause of mental retardation in children. The mechanisms underlying FASD are poorly understood. Thus, there is no effective treatment that targets the disorder.

Evidence from the human literature suggests that alcohol consumption during pregnancy results in adverse outcomes in fetuses and neonates (6). Fetal growth appears more sensitive to prenatal alcohol exposure than postnatal growth (7). Despite controlling for potential psychosocial confounders like tobacco use and partner violence, alcohol use during pregnancy appears to be associated with lower infant weight for age, height for age, and head circumference for age (8).

The animal model provides the opportunity to evaluate the effects of prenatal alcohol exposure on pups' birth weight in a controlled setting. Animal studies consistently suggest decreased fetal weight with prenatal alcohol exposure (9–11). The mechanism underlying FASDs is poorly understood. Thus, there is no effective treatment that targets the disorder. Novel therapies are critically needed to limit alcohol's adverse effects on the fetus exposed to alcohol during pregnancy.

Alcohol dose is also an important factor that plays a role in FASD. Effects of prenatal ethanol exposure on fetal growth, placentation, synaptic plasticity, and learning in mature offspring appear to be dose-dependent (12, 13). The threshold for eliciting subtle yet significant learning deficits in the offspring born to dams with prenatal alcohol exposure seems to be less than 30 mg/dl (3% alcohol diet), which equates to a blood ethanol concentration in the dam roughly equivalent to the consumption of 1–1.5 oz of ethanol per day in humans (12).

Maternal immune activation and elevation of cytokine levels (IFN-ɣ, IL-10, TNF-β, TNF-α, IL-15, IL-17) are associated with neurobehavioral impairment secondary to prenatal alcohol exposure (14–17). The ratio of TNF-α/IL-10 appears to be directly linked to the increased risk of having a child with FASD (18). Increased spontaneous production of interleukin-1β, interleukin-6, interleukin-12, and tumor necrosis factor-alpha by peripheral blood monocytes are seen in human adults with chronic alcoholism (19). Evidence suggests that inflammatory mediators may play a role in fetal growth restriction (20).

Inflammation is the principal response of the body to noxious insults. The initial response to injury involves specific cellular infiltrates and the release of pro-inflammatory lipid mediators, e.g., eicosanoids (prostaglandins, cytokines, leukotrienes, etc.), and immediately after, resolution occurs through anti-inflammatory and pro-resolution mediators (resolvins, protectins, epoxins, neuroprotectins, etc.). Both pro-inflammatory and pro-resolving mediators result from precursor polyunsaturated fatty acids (PUFA), principally arachidonic acid (AA), eicosapentanoic acid (EPA), and DHA (21). Alcohol is a noxious agent and, when ingested, initiates an inflammatory response characterized by the elaboration of reactive oxygen species and inflammatory mediators (22–26). Likely, the low birth weight associated with prenatal alcohol exposure results from unresolved inflammation and oxidative injury (27).

A preliminary study (27) from our group showed a significant decrease in mean pup weight with incremental prenatal alcohol exposure. The study utilized a control (pair-fed) and two alcohol (low-dose, i.e., 2.2 g/kg/day, and high-dose, i.e., 5.5 g/kg/day) treatment groups. Results showed a significant decrease in mean pup weight in the high-dose alcohol group when compared to the control group (1.91 g vs. 2.17 g, *p* = 0.007). However, the dose of alcohol used in the preliminary study was different from the current study.

Dietary lipids play a significant role in inflammation and its resolution. DHA (22:6n-3) is an n-3 polyunsaturated fatty acid (PUFA) and an essential precursor of the lipid mediators that help resolve inflammation (21, 28). We aim to utilize DHA as a novel nutritional intervention to determine if DHA supplementation in the pregnant rat will alleviate the effects of prenatal alcohol exposure on the fetus.

## Materials and methods

### Experimental design and procedures

The study utilized four groups of timed-pregnant Sprague-Dawley rats obtained from Charles River Laboratories and delivered to the Mott Center vivarium on gestational day (GD) 4. The animals were placed in individual housing to avoid confusion as to whom the pups belonged to in the event of premature delivery and to monitor food consumption per dam. Animals were provided with ad lib water and standard rat chow. The housing room was kept at a constant (22°C) temperature on 12 h dark and 12 h light schedule (lights off from 1,730 to 0530 h). Each pregnant rat was randomly assigned to the Control or Treatment (alcohol) group, as shown below.

Nine pregnant rats were assigned to Group 1 (Control) and five to each treatment group (Groups 2–4)—see Table 1. We randomly assigned three pregnant rats to a practice group to assess their tolerance to gavage feeding and alcohol, olive oil, and DHA.

**Table 1:** Groups and treatment categories after randomization of pregnant rats.

Group	Label	Treatment given
Group 1 (*N* = 9)	Control/Olive	0.8 ml olive oil + 1.8 ml Malto/dextrose
Group 2 (*N* = 5)	LD Alc/Olive	0.8 ml olive oil + 1.6 g/kg/day ethanol
Group 3 (*N* = 5)	HD Alc/Olive	0.8 ml olive oil + 2.4 g/kg/day ethanol
Group 4 (*N* = 5)	HD Alc/DHA	0.8 ml DHA + 1.6 g/kg/day ethanol

*N* = total number of pregnant rats (dams) per group; LD Alc, low dose alcohol; HD Alc, high dose alcohol; DHA, docosahexaenoic acid.

#### Group 1 (control; *N* = 9)

The pregnant rats were fed standard rat chow and water ad lib and served as the control. Group 1 was given two gavages; 1st gavage was pure olive oil (obtained from Essential Ingredients, Inc.), followed by distilled water plus maltose/dextrin. These two feeding sessions were about 1–2 h apart, starting approximately 10 AM each day, from gestational day 8 to day 19. Olive oil was used as a control lipid to DHA and given to all the rats in the study, except in the DHA supplementation group (Group 4). The volume of olive oil was equal to the average volume of DHA gavaged in Group 4 (0.8 ml). Maltose/dextrin solution served as the control for the caloric load of alcohol given to the treatment group, based on the caloric content of alcohol of 7 calories per gram and caloric content of maltose/dextrin of 95 calories per 25 g. A total of 9 rats were assigned to Group 1 to anticipate replacing rats in the treatment groups (Groups 2–4) that either resisted initial gavage feedings or died before the end of the study. It was estimated that 10% of the total rats (*N* = 15) in the treatment group might need replacement. There was no pair-fed control group because our previous study showed no significant difference in the ad-lib and pair-fed control groups. Similarly, we did not include a group given alcohol alone without lipid (DHA or olive oil) supplementation since our previous study showed that prenatal alcohol exposure alone resulted in low birth weight, brain weight in the fetus, and low placental weight (27).

#### Groups 2–4 (alcohol exposed groups)

Ethanol was administered to separate groups of pregnant rats based on alcohol dose or DHA supplementation. Each group received olive oil or DHA in 1st gavage and their daily alcohol dose in the 2nd gavage at 1–2 h apart, starting at approximately 10 AM from gestational day 8 to day 19. We used a 30% (v/v) ethanol solution made by diluting 30 ml of 99.5% ethanol solution (Spectrum Chemical MFG Corporation, New Brunswick, New Jersey) with 70 ml of distilled water. Peak blood alcohol levels at 0.5 h for the 2 g/kg dose and 3 g/kg doses have been reported in the literature as 91 ± 31 and 103 ± 29 mg%, respectively (29).

a) Groups 2 and 3 (Alcohol Dose Group: *N* = 5 per group)—Both groups received olive oil in their 1st gavage feeding and alcohol in 2nd gavage feeding. The alcohol doses were 1.6 g/kg/day (group 2) and 2.4 g/kg/day (group 3). For dosing, we used the initial weight of the dam as its weight throughout the experiment. Considering the concentration of the stock alcohol solution of 99.5% and specific gravity of alcohol of 0.7964 (density = 0.7964 g/ml), the volume of 30% alcohol needed for the 1.6 g/kg/day (group 2) and 2.4 g/kg/day (group 3) was calculated for each dam.
b) Group 4 (DHA supplementation)—A safety study in pregnant rats showed that a DHA dose of 1,250–2,500 mg/kg was safe and did not produce overt maternal toxicity. This dose did not result in changes in implantation losses, resorptions, live births, sex ratios, or fetal malformation (30). A daily dose of 1,250 mg/kg of DHA as DHASCO oil (from DSM Nutritional Products, Columbia, MD) was given as 1st gavage feeding. This group was also given daily 2.4 g/kg/day ethanol (30% v/v solution) as 2nd gavage feeding on gestational day 8 to day 19. DHASCO is a triglyceride extracted from the algae *Crypthecodinium cohnii*. DHASCO oil contains approximately 42.5% DHA and saturated and monounsaturated fatty acids. Group 3 served as the control group for group 4. To provide a dose of DHA of 1,250 mg/kg, assuming an initial weight of the dam of 0.4 kg (DHASCO oil contains 42.5% DHA or 42.5 g/100 ml or 425 mg/ml DHA), we calculated dose of DHA = 1,250 × 0.4 = 500 mg DHA, that was equivalent to 500/425 = 1.18 ml of DHA.

### Gavage feeding

The alcohol was administered directly into the dam's esophagus by a 3-in 18–20 gauge curved stainless steel, blunted needle designed especially for this purpose (Perfectum #7916 CVD). The animal was neither anesthetized nor tranquilized for this procedure not to confound the effects of prenatal alcohol exposure on the offspring by other drugs. Importantly, anesthetization or tranquilization was not required because this procedure was brief and tolerated reasonably well as performed by well-trained research team members. The rat was restrained by manual wrapping in a towel if needed, and the duration of restraint was kept as short as possible to minimize stress. The research team received prior formal animal training from the DLAR (Department of Laboratory Animal Research) on restraining a rat for the gavage feed. The volume and total daily ethanol doses were well-tolerated by the dams. The gavage was divided into two feedings that did not exceed the IACUC recommended maximum safety limit of 10 ml/kg per gavage feeding.

Each dam was monitored for labored breathing or any distress signs for 15 min after gavage feeding, as per the standard procedure.

### Saphaneous vein blood collection

This procedure was performed on GD 15 by holding the pregnant rat with gloves, leaving one hind limb exposed. The back of the leg was shaved off with an electric trimmer until the saphenous vein was visible. We used a small amount of water to keep the non-shaved hair away from the puncture site. We made a compression point at the base of the leg to make the saphenous vein bulge. We punctured the vein using a 20G needle and scooped the blood as it flowed out using sterile microvette tubes containing lithium heparin. After collecting 1 ml of blood, we held a clean compress on the puncture site to stop the bleeding.

### Euthanasia, blood and tissue harvesting

As the rat's typical duration of gestation is 21 days, we delivered the fetuses by gestational day 21 to prevent spontaneous delivery and breastfeeding by the pups. On GD 20, the dams were euthanized by rapid CO2 narcosis, followed by an assurance of death by cutting through the chest wall with a small scissor to produce a pneumothorax. Then the chest cavity was opened with the scissor, and blood was collected for cytokine analysis directly from the heart using a 21-gauge needle and 5 ml syringe, followed by cutting the heart to assure death. We collected at least 1–1.5 ml of blood in sterile microcuvette tubes containing lithium heparin. A laparotomy was performed, the uterine horns exteriorized, and the uterus opened. The immediate cessation of uterine blood flow and oxygen delivery to the fetus resulted in their rapid death. If the fetus began to breathe after its removal from the uterus, it was immediately euthanized by decapitation. The placenta was separated from the fetus, freed from the umbilical cord, deciduas, and fetal membranes blotted and weighed. Four pups were collected from each uterine horn and weighed.

### Processing blood sample

Immediately after collecting blood in heparinized microcuvette tubes, the blood samples were centrifuged for 10 min. The plasma was pipetted without disturbing the red and white blood cell layers. Plasma was stored at −80°C until the time of cytokine measurement by Luminex®-based analysis.

### Measurement of cytokines

Luminex® assays use antibody-conjugated bead sets to detect analytes in a multiplexed sandwich immunoassay format. Each bead in the set was identified by a unique content of two addressing dyes. A third dye was used to read out the binding of the analyte via a biotin-conjugated antibody and streptavidin-conjugated second step detector. Data was acquired on a dedicated flow cytometry-based Luminex platform (31).

### Statistical analysis

The pups were weighed at birth, and the mean and placental weights (g) between groups were compared by one-way analysis of variance (ANOVA) with *post hoc* Duncan's test. A test for homogeneity was performed using Levene's test for equality of variances. The Levene's test showed equal variances in the groups.

A *p*-value of ≤0.05 was considered a level of statistical significance. Statistical analysis was performed using SPSS Version 28.

## Results

### Pup body weight

**Figure 1:**
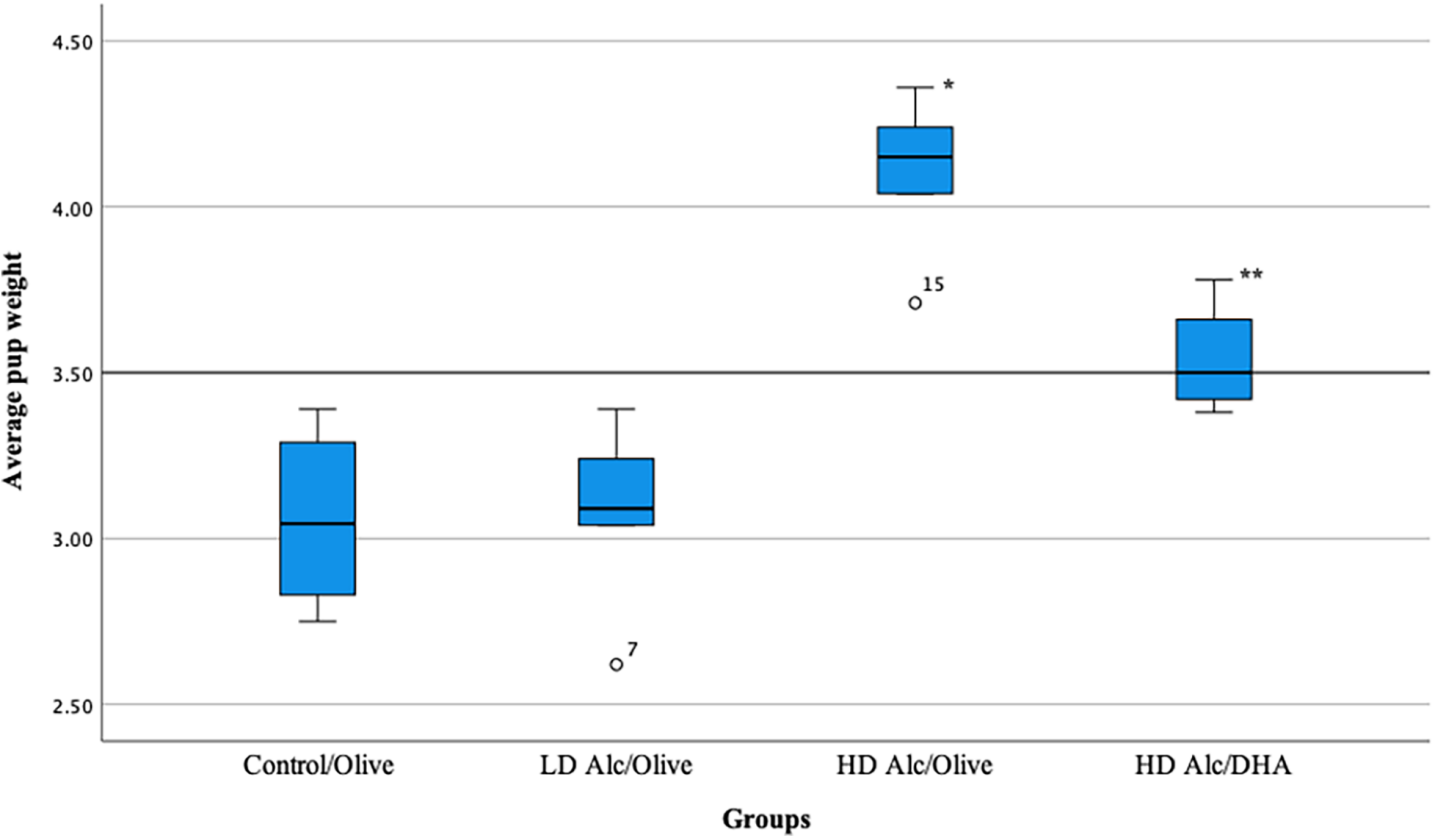
Box plot showing the average pup weight in different groups. LD Alc, low dose alcohol; HD Alc, high dose alcohol; DHA, docosahexaenoic acid. *p = 0.05 when compared to control/olive group, LD Alc/Olive group, or HD Alc/DHA group. **p = 0.05 when compared to control/olive group, LD Alc/Olive group, or HD Alc/olive group.

In Table 2 and Figure 1, the mean birth weight of pups was significantly higher in pups prenatally exposed to HD alcohol/olive oil when compared to the control/olive oil group (4.10 g vs. 3.06 g, *p* = 0.05). Similarly, the mean birth weight of pups prenatally exposed to HD alcohol/DHA was significantly higher (3.54 g vs. 3.06 g, *p* = 0.05) when compared to the control/olive oil group. The mean birth weight of pups was also significantly higher in pups prenatally exposed to HD alcohol/olive oil when compared to the HD alcohol/DHA group (4.10 g vs. 3.54 g, *p* = 0.05). Similarly, the mean birth weight of pups was also significantly different between LD alcohol/olive oil group and HD alcohol/olive oil group (3.08 g vs. 4.10 g, *p* = 0.05) or HD alcohol/DHA group (3.08 g vs. 3.54 g, *p* = 0.05). However, there was no significant difference in the mean birth weight of pups in the LD alcohol/olive oil group when compared to the control/olive oil group (3.08 g vs. 3.06 g, *p* > 0.05). The pregnant rats' mean weight was not significantly different between groups at GD 20.

**Table 2:** Mean birth weight (± SD) of rat pups in control, alcohol/olive, and alcohol/DHA categories (comparison by one-way ANOVA with Duncan's test).

Group	*N*	*n*	Mean wt (g)	SD	95% confidence interval	
					Lower bound	Upper bound
Control/Olive	6	48	3.06	0.25	2.80	3.32
LD Alc/Olive	5	38	3.08	0.29	2.72	3.44
HD Alc/Olive	5	40	4.10*	0.25	3.79	4.41
HD Alc/DHA	4	32	3.54**	0.17	3.27	3.82

*N* = total number of dams per group (final number of dams was less than planned dams per group due to death of some dams during the study); *n* = total number of pups per group (8 pups per dam, 1 dam in LD Alc/Olive only had 6 pups); LD Alc, low dose alcohol (1.6 g/kg/day); HD Alc, high dose alcohol (2.4 g/kg/day); DHA, docosahexaenoic acid; SD, standard deviation.

* *p* = 0.05 when compared to control/olive group, LD Alc/Olive group, or HD Alc/DHA group.

** *p* = 0.05 when compared to control/olive group, LD Alc/Olive group, or HD Alc/olive group.

In Table 3, the mean placental weight was significantly higher in all treatment (alcohol) groups. The mean placental weight was significantly lower in the control/olive oil group when compared to LD alcohol/olive oil group (0.49 g vs. 0.59 g, *p* < 0.05), to HD alcohol/olive oil group (0.49 g vs. 0.57 g, *p* < 0.05) or to HD alcohol/DHA group (0.49 g vs. 0.58 g, *p* < 0.05).

**Table 3:** Mean placental weight (± SD) in control, alcohol/olive, and alcohol/DHA categories (comparison by one-way ANOVA with Duncan's test).

Group	*N*	*n*	Mean placental wt (g)	SD	95% confidence interval	
					Lower bound	Upper bound
Control/Olive	6	48	0.49	0.08	0.46	0.51
LD Alc/Olive	5	38	0.59*	0.12	0.55	0.63
HD Alc/Olive	5	40	0.57*	0.07	0.55	0.59
HD Alc/DHA	4	32	0.58*	0.10	0.55	0.61

*N* = total number of dams per group (final number of dams was less than planned dams per group due to death of some dams during the study); *n* = total number of pups per group (8 pups per dam, 1 dam in LD Alc/Olive only had 6 pups); LD Alc, low dose alcohol (1.6 g/kg/day); HD Alc, high dose alcohol (2.4 g/kg/day); DHA, docosahexaenoic acid; SD, standard deviation.

* *p* < 0.05 when compared to control/olive group.

### Cytokine analysis

Higher pro-inflammatory cytokines (principally Interleukin -1 beta and Interleukin -12p70) were found in the alcohol-exposed pregnant rats (Table 4). However, the difference in the level of cytokines was not statistically significant between different treatment categories due to the large variance and small sample size.

**Table 4:** Mean cytokine values in different treatment categories.

Cytokines		Control/Olive	LD Alc/Olive	HD Alc/Olive	HD Alc/DHA
Day 15 IL1 beta	Mean (±SD)	61.0 (±78.6)	22.9 (± 29.9)	109.9 (±60.5)	105.6 (±71.1)
	*N*	5	5	4	3
Day 15 IL12p70	Mean (±SD)	52.6 (±40.8)	344.9 (±549.8)	68.5 (±29.3)	87.4 (±16.6)
	*N*	5	3	3	2
Day 15 TNF-alpha	Mean (±SD)	0	2.3 (±5.0)	1.7 (±3.8)	2.1 (±4.3)
	*N*	6	5	5	4
Day 20 IL1 beta	Mean (±SD)	2.8 (±4.5)	0	1 (±1.4)	10.5 (±21.0)
	*N*	5	5	5	4
Day 20 IL12p70	Mean (±SD)	42.4 (±14.2)	138.2 (±195.5)	16 (±32.1)	41.7 (±10.6)
	*N*	4	2	4	2

*N* = total number of dams per group (final number of dams was less than planned dams per group due to death of some dams during the study); LD Alc, low dose alcohol (1.6 g/kg/day); HD Alc, high dose alcohol (2.4 g/kg/day); DHA, docosahexaenoic acid; SD, standard deviation; IL, interleukin.

## Discussion

Globally, it has been estimated that about 10% of women in the general population consume alcohol during pregnancy, and 1 in 67 women deliver a child with FAS (4). Another report estimates the pooled prevalence of FAS and FASD in the United States is about 2 per 1,000 and 15 per 1,000, respectively, among the general population (3).

The overall objective of this work was to evaluate DHA supplementation as a potentially novel therapy to ameliorate the adverse effects of prenatal alcohol exposure in FASDs. However, incidental to our principal objective was the observation that supplementation of alcohol with both olive oil and DHA significantly increased birth weight in alcohol-exposed pups. The positive impact of DHA supplementation on birth weight in prenatal alcohol exposure is consistent with one report of n-3 PUFA supplementation and improved body weight of pups (10); however, the report utilized both prenatal and postnatal supplementation strategies with n-3 PUFA.

Our previous study did not show any difference in placental weight in the alcohol-exposed vs. control fetuses (27). Additionally, a decrease in placental weight has been reported in different animal models of prenatal alcohol exposure (32, 33). However, the results of this report showed otherwise, i.e., an increase, rather than decrease, in placental weight in alcohol-exposed fetuses, which has been reported in other studies (34–36). The increase in placental weight likely represents a compensatory response to the alcohol-induced insult. Our study is unique as it utilized dietary lipids to mitigate the potential adverse effects of alcohol on fetal weight. Olive oil has been shown to reduce placental stress in gestational diabetes (37). Similarly, dietary supplementation with n-3 PUFA in pregnant women increases DHA levels, reduces placental oxidative stress, and enhances placental and fetal growth (38, 39). Thus, the variations in placental weight outcome warrant further study.

In humans, high alcohol intake during pregnancy has been associated with DHA deficiency in the maternal plasma (40). One mechanism may be that DHA is esterified and excreted in the urine as an ethyl ester (41). DHA has antioxidant, anti-inflammatory, and pro-resolving properties (42) and is preferentially transferred across the placenta from the mother to the fetus (43). Thus, supplementing the alcoholic pregnant woman with DHA may be a plausible intervention to reduce inflammation-driven injury from alcohol in the fetus and ameliorate the adverse effect of alcohol on the infant's birth weight. This report is part of a large study whose primary aim was to determine the protective effect of DHA in the offspring of pregnant rats prenatally exposed to alcohol. As a lipid control for DHA, olive oil was used, which led to the unexpected finding of the salutary effect of both DHA and olive oil on the birth weight of the pups. However, the weight gain with DHA was not as high as with olive oil supplementation in the high-dose alcohol-exposed groups. Although olive oil can have a positive effect on pups' weight because of its caloric content of 8.5 calories per gram, it should be noted that in the study, olive oil's potential positive effect on pups' birth weight was not evident in the low dose alcohol/olive oil group when compared to the control/olive group and was significantly lower when compared to the alcohol/DHA group. Thus, olive oil likely did not interfere significantly with the ameliorative property of DHA on birthweight.

Olive oil has been studied in varied clinical conditions to mitigate the effects of oxidant stress, including aging (44, 45). Olive oil benefits have been suggested in preventing cardiovascular diseases, improving the gut microbiota, and mitigating inflammation, including inflammatory bowel diseases and psoriasis (46–48). Olive oil intake also has some beneficial effects on colorectal cancer prevention (49). Hydroxytyrosol (HT) is a primary polyphenol in olive oil with anti-inflammatory and neuroprotective properties (50, 51). HT has both lipophilic and hydrophilic properties, allowing it to be absorbed readily and exhibit cytoprotective properties by scavenging free radicals and limiting inflammation (51). Maternal HT supplementation has been shown to enhance mean birth weight in animal studies (52). However, current evidence from published literature is insufficient to suggest any such beneficial potential of olive oil in the setting of FASD.

Since this salutary effect of the dietary lipids on the pups' birth weight was more prominent in high-dose (2.4 g/kg/day) alcohol-exposed groups, we speculate that these findings could have resulted from the caloric contribution from high-dose alcohol exposure and dietary lipids. Furthermore, more calories were delivered to the dams in the high-dose alcohol group (2.4 g/kg/day) compared to the low-dose alcohol (1.6 g/kg/day) group, which may account for the differential weights between the 2 groups. Likewise, the DHA loss through alcohol esterification may also reduce its caloric potential and explain the lower weight gain with alcohol/DHA compared to alcohol/olive oil (41).

It is also possible that this may be the effect of increased visceral fat biosynthesis and accumulation in response to the interaction of alcohol and high saturated fat content in olive oil (53). It is also likely that DHA has a protective effect on different organs (42, 54–56), which may have contributed to the positive impact of both olive oil and DHA on the birth weight of alcohol-exposed pups.

Our study is significant since it provides evidence that DHA and olive oil may have a therapeutic potential to mitigate the adverse effects of low birth weight in offspring prenatally exposed to alcohol. Additionally, these dietary lipids may have a positive effect on the placental growth.

Cytokine analysis showed a trend toward increasing maternal serum IL-1 and IL-12 with alcohol exposure. However, the difference was not statistically significant, likely due to the large variance in the sample mean concentrations and needs to be studied further using a larger sample size.

We conclude that DHA and olive oil supplementation in alcohol-exposed pregnant rats significantly increased the birth weight of their pups and the placental weight of the fetus, although the mechanism of this effect remains to be determined. In translating the results of this animal study to humans, supplementing alcohol-abusing pregnant women with dietary lipids (DHA and olive oil) may improve the birth weight of their newborn infants. However, prenatal alcohol exposure, *per se*, has been linked to future risk of obesity and related cardiometabolic consequences (diabetes, hypertension) in children with FASD (57–59), and likely represents the fetal basis of adult diseases. Thus, supplementing prenatal alcohol exposure with lipids to improve birth weight may unknowingly contribute to this potential long-term effect.

Finally, our study design did not include a pure alcohol group as a control group since the negative effect of alcohol on birthweight is already well known and has been reported not only by us but by other investigators, as well, in both human and animal studies (60–63). However, the absence of a pure alcohol-supplemented group could be a limitation of this study.

The data supporting our study's findings are available from the corresponding author, [DY], upon reasonable request.

## Data Availability

The raw data supporting the conclusions of this article will be made available by the authors, without undue reservation.
